# Coping and Co-Occurrence of Gaming Disorder and Substance Use in Recovering Substance Users

**DOI:** 10.3390/jcm11247370

**Published:** 2022-12-12

**Authors:** Tyrone L. Burleigh, Mark D. Griffiths, Alexander Sumich, Grace Y. Wang, Daria J. Kuss

**Affiliations:** 1 International Gaming Research Unit, Nottingham Trent University, Nottingham NG1 4FQ, UK; 2 Centre of Excellence in Responsible Gaming, University of Gibraltar, Gibraltar GX11 1AA, UK; 3 NTU Psychology, Nottingham Trent University, Nottingham NG1 4FQ, UK; 4 School of Psychology and Wellbeing, University of Southern Queensland, Darling Heights, QLD 4350, Australia; 5 Centre for Health Research, University of Southern Queensland, Darling Heights, QLD 4350, Australia

**Keywords:** gaming disorder, substance use disorder, co-occurrence, addiction, coping, substance abstinence

## Abstract

Background: There are a wide range of negative effects associated with both substance use disorders and behavioural disorders and their co-occurrence. Understanding the way in which at-risk populations (e.g., substance-abstinent users) interact with potentially addictive behaviours (e.g., gaming) and substance use—while navigating life stressors through differing coping styles—can inform preventative strategies. Methods: Therefore, the present study investigated 64 clinical participants and 138 general population participants. Each cohort was required to complete a battery of psychometric scales exploring problematic behaviours, problematic substance use, co-occurrence, and coping styles. Additional exploratory direct comparisons of gamers in the clinical cohort and gamers in the general cohort were carried out. Results: The study’s findings suggest that gamers from different populations (i.e., general and clinical) share similar at-risk behaviours. These problematic behaviours were more pronounced among abstinent substance use gamers, and more specifically among poly-substance use gamers. Conclusions: The findings of the present study add to the literature and suggest that dysfunctional coping style and the co-occurrence of problematic behaviours may have an impact on the assessment and potential treatment of substance abstinent gamers. The findings offer support for an integrated treatment approach, wherein both substance use and the other problematic behaviours (e.g., gaming) are considered in tandem.

## 1. Introduction

Research into substance use disorders (SUDs) has demonstrated a wide range of negative effects on neurocognitive functions and psychological wellbeing [1]. Similarly, specific potentially addictive behavioural disorders (e.g., problematic online gaming) are now understood as mental health disorders due to evidence associating excessive use with adverse changes in brain function and psychological wellbeing [2]. Consequently, Internet Gaming Disorder (IGD) was included in the Diagnostic and Statistical Manual of Mental Disorders (DSM-5) as a tentative behavioural addiction warranting further investigation [3]. Furthermore, the World Health Organization (WHO) has now officially recognized Gaming Disorder (GD) in the latest eleventh revision of the International Classification of Diseases (ICD-11) [4].

Prior to the inclusion of IGD in the DSM-5 and GD in the ICD-11, several other terms were used to describe problematic videogaming including (but not limited to) videogame addiction, pathological videogaming, gaming use disorder, and gaming use dependency [5]. Furthermore, some scholars have included problematic online gaming within the umbrella terms of internet addiction, problematic internet use, and pathological internet use [6]. In order to maintain consistency throughout the present paper, the term ‘problematic gaming’ will be used to describe a range of similar and/or overlapping addictive, compulsive, and/or subclinical behaviours related to gaming. When referring to clinically defined cases, the term ‘GD’ will be used, in line with the ICD-11. Furthermore, in relation to other potentially addictive behaviours or substance use, the term ‘problematic’ will be used to describe sub-clinical conditions that do not fully meet all the criteria in the DSM-5 or ICD-11 (e.g., ‘problematic gambling’), while the term ‘disordered’ will be used to describe clinical conditions that meet the requisite criteria in the ICD-11 (e.g., gambling disorder).

### 1.1. Gaming Disorder and Comorbid Disorders

Several studies have reported an association between GD and mental health disorders, such as anxiety [7], depression [8], and personality disorders [9]. Similarly, the literature suggests that disordered gaming can co-occur with other problematic, addictive, and disordered behaviours [10]. It is worth noting that not all problematic behaviours are recognized as disorders. Instead, they may be considered as ‘potential’ behavioural addictions, such as shopping addiction (often referred to as ‘compulsive buying’), social media addiction, and work addiction [10]. These behaviours (including GD) share a conceptual basis with substance use disorders. However, the use of ‘addiction’ terminology to describe these behaviours is heavily debated in the literature. For example, some scholars believe that there has not been a sufficient scientific basis established for the GD diagnosis [11]. It has been suggested that the excessive ‘disorder-like’ activities which relate to these disorders may instead be a reflection of investment rather than clinically disordered behaviour [12,13]. For example, one specific symptom of disordered behaviour is salience (i.e., being consistently preoccupied with the disordered behaviour), which may not be accurately reflected in the context of gaming. This is due to the time investment discussing and optimizing the game experience. Consequently, there are claims that the diagnosis has poor specificity which could lead to inaccurate diagnosis of approximately one-third of gamers [13]. Nevertheless, evidence has consistently indicated that disordered or problematic gaming is associated with an increased risk of psychopathology (e.g., depression, anxiety [14], and disordered substance use [10]). Consequently, it has been posited that these increased risks may instead be a result of, or partly attributed to, maladaptive coping strategies [15] or other psychopathological conditions. 

### 1.2. Coping

Coping refers to the cognitive and behavioural responses that process and manage stressful events and emotions [16]. Several studies have considered the relationship between coping and gaming [10]. The Brief COPE [17] has been used, which is based on Lazarus and Folkman’s [18] transactional model of stress and Carver and Scheier’s [19] behavioural self-regulation model. The domains assessed are emotion-focused coping and problem-focused coping. Problem-focused coping involves efforts to modify the stressful event and includes strategies, such as producing options to solve the problem, evaluating the pros and cons of the options, and implementing steps to solve the problem. Emotion-focused coping is typically defined as the attempt to manage emotional distress that is associated with a stressful event [18]. There are a broad range of strategies which fall under this domain, such as the use of humour, positive reframing of the distressing situation, acceptance, seeking social support, and/or the use of religion. However, emotion-focused coping has also included strategies such as denial, venting and/or fixating on negative emotions [17]. Therefore, it is not surprising that the effectiveness of emotion-focused coping can vary depending on which strategy is employed [20].

As a result of these (somewhat) opposed groupings, it appears that engaging in emotion-focused coping can be considered maladaptive. While Carver et al. [17,20] did not utilise specific domains of coping, the domains of being emotion-focused and problem-focused have been used in conjunction with their measures in previous research [15,21]. Therefore, the present study adopts the definitions of previous papers which defines emotional coping as a specific subset of emotion-focused strategies which actively engage with distress (e.g., using humour) as opposed to avoiding the stressful event in a passive and static way (e.g., denial) [15,21,22]. In line with previous literature, the present study also uses a third domain of coping, which focuses on dysfunctional coping [21]. Within the scope of the present paper, dysfunctional coping is defined as disengaging with a stressful event in an avoidant manner through avoidant coping strategies such as denial, substance use, and self-distraction [6,21].

Some evidence suggests that individuals may play videogames excessively to cope with everyday stressors and to regulate their emotions [23]. While using videogames to de-stress and ‘escape’ is not maladaptive itself, for some gamers, excessive game play has predicted maladaptive escapism or self-distraction (e.g., turning to work or other activities to take their mind off things) as a coping strategy [24], which can result in poorer mental health outcomes and exacerbate excessive gameplay, creating a cycle of reciprocity [10]. Moreover, this type of avoidant coping has been reported in both the gaming literature and the substance use literature [10,25]. These dysfunctional strategies are often overly relied upon among adolescents [21] and could carry through into emerging adulthood and adulthood [26], resulting in poor emotional regulation [27].

### 1.3. Substance Use and Gamers

Parallels exist between the way gamers and substance users regulate their emotions [27]. It has been suggested that this could be due to the aforementioned similarities in the common coping strategies employed by each group, such as behavioural disengagement (i.e., self-distraction) [10]. This association may be explained through the self-medication hypothesis; i.e., within addiction-related disorders, substances (or specific behaviours) can be used to overcome distressing affective states and unpleasant symptoms [28]. This behaviour is indicative of a dysfunctional coping strategy, which has been suggested to play a role in the development of co-occurring behaviours within disordered gaming [10] and substance use [29]. While some literature has suggested that dysfunctional coping strategies play a role in disordered gaming and substance use, there is a dearth of literature that considerers clinical substance users who may also engage in problematic gaming behaviours [10,29]. This is problematic because the literature consistently provides evidence on the similarities between problematic/disordered behaviours, substance use disorders, and the coping strategies they employ along with the potential of their co-occurrence [30,31,32,33]. Moreover, the evidence suggests that when individuals are faced with distressing situations and engage in the use of dysfunctional strategies, symptoms of disordered behaviour or substance use increase—more so than the engagement in either one on their own [21,25]. Gaming disorder, similar to other addictions, including other behavioural addictions (e.g., gambling disorder), can co-occur with other problems [34]. Therefore, disordered gaming may also interact and/or co-occur with other conditions such as disordered substance use.

### 1.4. Replacement, Co-Occurrence and the Cycle of Reciprocity

Within the present study, co-occurrence refers to when two or more potentially addictive behaviours (behavioural and/or substance) are engaged in concurrently. It has been suggested that co-occurrence of problematic or disordered behaviour and substance use can be attributable to shared psychological mechanisms underlying these behaviours (e.g., the aforementioned coping mechanisms; [15,21]) in the development and maintenance of co-occurrence between and within problematic behaviours and substance use [10].

Different theories have been put forward regarding how various potentially addictive behaviours, such as gaming and substance use, may co-occur with one another. For example, replacement theory suggests that substances or behaviours have the potential to replace each other [35]. This theory aligns with the behavioural self-regulation model of coping [19] and the self-medication hypothesis [28]. Behavioural self-control theory suggests that an individual engages in a feedback loop of self-regulation (i.e., a coping strategy) when trying to reach a goal. In doing so, an individual monitors their progress and adjusts their behaviour accordingly to improve their efforts. If individuals begin to use substances or engage in behaviours to alleviate the affected mood state, this may result in the development of a dysfunctional coping strategy. As a result, when they are confronted with the stressful event, they may return to the substance or behaviour that alleviated the last mood state effectively (i.e., self-medication [28]). However, when ending the disordered behaviour in question (either voluntarily or involuntarily), an individual may become vulnerable to other potentially disordered behaviours to explore other avenues of satisfying their needs [35]. Therefore, replacement theory posits that disordered behaviours (i.e., behavioural or substance-related) serve a specific function (e.g., escape, coping, relaxation), and individuals who cease these behaviours will seek to replace them [28]. Indeed, empirical literature has demonstrated a wide variety of behaviours or substances which some individuals can become dependent on (e.g., alcohol), or repeat excessively (e.g., gaming) when abstaining from disordered substance use (i.e., reciprocity [35]).

It has been suggested that the abstaining individual may then seek to substitute these behaviours with an activity they perceive as being less detrimental than the original disordered behaviour. To the abstaining individual, the replacement behaviour may appear adaptive and reasonable. However, it may be disadvantageous depending on which behaviour is chosen [35], indicating there is an at-risk group of individuals who are abstaining from problematic or disordered behaviours/substance use. Indeed, it has been shown that substance users, when abstaining from the primary substance, may develop other behaviours that fill that dysfunctional process. For example, substance users may replace illicit substances with cigarette smoking or sexual behaviour, behaviours that they may feel are less detrimental than illicit substances [36]. It has also been highlighted that individuals with disordered substance use believe that internet use is a safe substitute to utilize in their repertoire of existing coping strategies [35]. Likewise, the literature suggests that other problematic behaviours can also be utilized in similar way [21]. However, there are few studies which explore the role of gaming in the context of abstinent substance users [10]. This is especially important given that gaming has been found to be significantly associated with a number of potentially addictive behaviours and substances [31,32].

In a recent epidemiological study on the co-occurrence of substance use and other potentially addictive behaviours, it was found that there were large overlaps between the co-occurrence of disordered substance use and/or behaviours [37]. More specifically, Kotyuk et al. [37] found that alcohol use and cigarette smoking were associated with problematic gaming among a large sample of emerging adults. Additionally, in a recent systematic review, Burleigh et al. [10] also reported similar overlaps of substance use and behaviours among adolescents, emerging adults, and adults. Indeed, the evidence supports the potential for disordered behaviours to increase the propensity of other related problematic behaviours. Accordingly, the overlap of two or more problematic behaviours may then create a cycle of reciprocity [38,39,40], wherein mutual exacerbation occurs. Furthermore, individuals who experience multiple problematic and/or disordered behaviours have been shown to be at higher risk of poorer mental health (e.g., depression) and physical health [26,40,41].

Therefore, considering the co-occurrence of problematic behaviours and substance use is relevant for a number of reasons. Firstly, it is rare that clinical symptomology emerges on its own, as it can be a consequence of a collection of risk factors [42]. Secondly, being aware of how co-occurring problematic and disordered behaviours react and enforce facets of a primary problematic or disordered behaviour has an important clinical significance [10]. For example, disordered gaming may mask problematic substance use within an individual, therefore hindering diagnostic assessment. Alternatively, disordered gaming may increase problematic substance use, causing symptoms of both to alternate, which can impact treatment efficacy [43]. Therefore, co-occurrence has the potential to impact the onset, course, and outcomes of treatment interventions [10]. Thirdly, understanding these associations will aid future assessment and treatment paradigms through the consideration of both the presenting primary disordered (or problematic) behaviour (or substance use), and any potential co-occurring behaviours or substance use, which may enforce a cycle of reciprocity [44,45]. Finally, being aware of these co-occurrences will also help inform prevention strategies [46].

### 1.5. The Present Study

While the understanding of the nuanced issues of diagnosis and case formulation requires evaluation and testing, there is currently a dearth of literature which has investigated the coping styles and the co-occurrence of problematic/disordered gaming with other potentially addictive substance use and behaviours within clinical populations in treatment. The literature suggests that individuals in clinical populations who experience a co-occurring behavioural, or substance use disorder (or problematic use) tend to have poorer functioning and treatment outcomes in comparison to non-clinical populations. This mirrors findings within disordered gaming because disordered gamers also experience poorer functioning and treatment outcomes [47,48,49]. Despite the acknowledged association between gaming and other addictive behaviours, it is unclear how the strength of this association is affected by an individual’s severity of addiction and coping style. According to developmental perspectives, addiction should be viewed along a continuum, ranging from functional use (i.e., no problems) to dysfunctional pathological use. Therefore, understanding the dynamic changes in co-occurring addictive behaviours from adaptive to pathological use has important implications for clinical assessment and treatment planning, including being able to evaluate which treatment would be appropriate based on the individual’s behaviour patterns and where they fall on the continuum. 

In the present study, co-occurring problematic behaviour and substance use in clinical (i.e., substance users in treatment) and a non/sub-clinical (i.e., general population) sample of gamers and non-gamers were compared and contrasted, and the effect of coping strategies on gaming and its co-occurrence with substance use and other problematic behaviours was explored. Accordingly, the following hypotheses were formulated:

**Hypothesis** **1** **(H1).** *Gamers will exhibit more severe co-occurring problematic behaviours among both clinical and non-clinical samples when compared to non-gamers, with co-occurring substance use exacerbating disordered gaming among the clinical sample*.

**Hypothesis** **2** (**H2).** *The severity of gaming among both clinical and non-clinical groups will be positively associated with dysfunctional coping styles, but negatively associated with problem-focused coping styles*.

## 2. Materials and Methods

### 2.1. Participants and Procedure

Clinical data were collected from October 2019 to January 2020 at a rehabilitation centre in New Zealand from patients who were undertaking treatment for their disordered substance use. The inclusion criteria were: (i) being aged 18 years and over, and (ii) having previously or currently experienced disordered substance use. Each participant was given a $20 NZ voucher for participating. The clinical sample comprised 64 participants, comprising 49 men (M_age_ = 35.73 years; *SD* = 10.95) and 15 women (M_age_ = 37.14 years; *SD* = 10.48), aged between 22 and 88 years (M_age_ = 36.06 years; *SD* = 10.77). Participants that listed that they played videogames were classified as gamers. Additional sociodemographic information can be seen in Table 1. 

In regard to substance use details, the average age of onset for the primary substance use was 13.31 years (*SD* = 2.76). Out of the 64 substance users surveyed, 60 were currently abstinent. Abstinence ranged between seven days to ten years (M_days_ = 257.32; *SD* = 496.10). The remaining four participants (who were new to the centre) had been abstinent less than a day. In addition, 45.3% reported an accompanying mental health disorder (e.g., depression, anxiety) and of those, 21.9% had two or more comorbid mental health disorders. Additional substance use information can be found in Table 2.

Data from the control sample were collected from Auckland, New Zealand (between September 2019 to March 2020) which is located in a similar geographical region to the rehabilitation centre. Flyers and electronic advertisements (e.g., SONA platform) were used around a university campus. Students were offered the chance to win a $50 NZ shopping voucher. The inclusion criteria for this sample were: (i) being aged 18 years and over; and (ii) currently residing in New Zealand. The control group sample comprised 138 participants, including 72 males (M_age_ = 26.86 years; *SD* = 9.83) and 59 females (M_age_ = 27.49 years; *SD* = 9.07 years), aged between 18 and 65 years (M_age_ = 26.54 years; *SD* = 9.47). Additional sociodemographic information can be seen in Table 3. All participants were given information prior to the study, including data use, potential risks, and benefits, along with their right to withdraw from the study at any time. The study was approved by the research team’s two university ethics committees.

### 2.2. Measures

#### 2.2.1. Nine-Item Internet Gaming Disorder Scale—Short Form (IGDS9-SF)

The nine-item IGDS9-SF [50] was used to assess the severity of GD symptoms (over the past 12 months) by examining an individual’s offline and online behaviours. Items of the IGDS9-SF include: *“Do you systematically fail when trying to control or cease your gaming activity?”* and *“Have you jeopardized or lost an important relationship, job or career opportunity because of your gaming activity?”*. Participants respond to each item on a five-point scale from 1 (*Never*) to 5 (*Very often*). The final GD score is calculated by summing up the individual’s answers and ranges from 9 to 45, with higher scores indicating higher severity of gaming disorder behaviours. The scale has been shown to be a reliable measure with a Cronbach’s α of 0.88 [50]. In the present study, the general cohort and clinical cohort scale scores showed very good reliability with a Cronbach’s α of 0.89 and 0.94, respectively.

#### 2.2.2. Problem Gambling Severity Index (PGSI)

The nine-item PGSI [51] was used to assess gambling behaviours over the past 12 months (e.g., *“Have you gone back on another day to try to win back the money you lost?”*). Participants respond to items on a four-point scale ranging from 0 (*Never*) to 3 (*Always*). The total score is obtained by summing up each of the answers given and can range from 0 to 27, with higher scores indicating higher gambling severity. The final score is then related to one of four domains: non-problem gambler = 0; Low-risk gambler = 1–2; Moderate-risk gambler = 3–7; Problem gambler = 8 or above. This has been shown to be a reliable scale with a Cronbach’s α of 0.76 [51]. In the present study, the general cohort and clinical cohort scale scores showed excellent reliability with a Cronbach’s α of 0.96 and 0.98, respectively.

#### 2.2.3. Nine-Item Internet Disorder Scale—Short Form (IDS9-SF)

The nine-item IDS9-SF [52] was used to assess problematic internet use behaviours over the past 12 months (e.g., *“Do you feel more irritability, anxiety and/or sadness when you try to either reduce or stop using the internet?”*). Responses are scored on a five-point scale ranging from1 (*Never*) to 5 (*Very often*). The final score is calculated by adding each item score which gives a total score ranging from 9 to 45. Higher scores indicate a higher severity of disordered internet use. The IDS9-SF has been shown to be a reliable scale with a Cronbach’s α of 0.96 [52]. In the present study, the general cohort and clinical cohort scale scores showed excellent reliability with a Cronbach’s α of 0.92 and 0.95, respectively.

#### 2.2.4. The Bergen Social Media Addiction Scale (BSMAS)

The BSMAS [53] is an adapted version of the Bergen Facebook Addiction Scale (BFAS; [54]) and includes six items assessing addictive social media use (e.g., *Facebook, Instagram, Twitter*) over the past 12 months. Each item reflects a core addiction element (e.g., withdrawal: *“How often have you become restless or troubled if you have been prohibited from using social media?”*), is scored on a five-point scale ranging from 1 (*Very rarely*) to 5 (*Very often*) and can have a total score between 6 and 30. A higher score indicates a greater risk of being addicted to social media use. The BSMAS has very good reliability (Cronbach’s α = 0.88) [53]. In the present study, the general cohort and clinical cohort scale scores showed excellent reliability with a Cronbach’s α of 0.90 and 0.93, respectively.

#### 2.2.5. Bergen-Yale Sex Addiction Scale (BYSAS) 

The six-item BYSAS [55] was used to assess participants’ problematic sexual activity over the last 12 months (e.g., *“How often … have you spent thinking about sex or masturbation?”*). Each response is scored on a five-point scale, with scores ranging from 0 (*Very rarely*) to 4 (*Very often*). To obtain the total score, the scores on each item are summed. The total score can range from 0 to 24, with a higher total score indicating a greater risk of addictive sexual behaviour. The BYSAS has been found to be a reliable scale with a Cronbach’s α of 0.82 [55]. In the present study, the general cohort and clinical cohort scale scores showed excellent reliability with a Cronbach’s α of 0.90 and 0.92, respectively.

#### 2.2.6. Bergen Shopping Addiction Scale (BSAS)

The seven-item BSAS was used to assess for problematic shopping behaviour over the past 12 months [56]. Participants respond to each item (e.g., *“I think about shopping or buying things all the time”*) on a five-point Likert scale from 0 (*completely disagree*) to 4 (*completely agree*). The final score is calculated by summing up the individuals’ answers and ranges from 0 to 28, with higher scores indicating greater risk of shopping addiction. The BSAS has been found to be a reliable scale with a Cronbach’s α of 0.87 [56]. In the present study, the scale showed excellent reliability in both the general cohort and the clinical cohort (Cronbach’s α = 0.89 and 0.93).

#### 2.2.7. Cigarette Dependency Scale-5 (CDS)

The five-item CDS-5 [57] was used to assess the degree to which participants were dependent on cigarettes. Each item is scored on a five-point scale and assesses their cigarette use (e.g., *“Please rate your addiction to cigarettes on a scale of 0 to 100?”*) and habits (e.g., *“Usually, how soon after waking up do you some your first cigarette?”*). Questions 1 to 3 are open questions (e.g., “On average, how many cigarettes do you smoke per day?”) where a participant can write their response. The response is then converted into a five-point scale (e.g., “8 cigarettes per day” equates to a score of 2 [6–10 cigarettes per day]). Questions 4 and 5 require typical responses with scores ranging from 1 (*Totally Disagree*) to 5 (*Fully Agee*). Questions 3 and 4 are both reverse coded, where the lower point is scored as 5 and the higher point is scored as 1 (e.g., *“For you, quitting smoking would be “Impossible”* [5] to *“Very Easy”* [1]). The CDS-5 has been found to be a reliable scale with a Cronbach’s α of 0.83 [57]. In the present study, the general cohort and clinical cohort scale scores showed good reliability with a Cronbach’s α of 0.75 and 0.76, respectively.

#### 2.2.8. Alcohol Use Disorder Identification Test (AUDIT)

The ten-item AUDIT [58] was used to assess alcohol consumption, drinking behaviours, and alcohol-related problems over the past 12 month (e.g., *“How often do you have six or more drinks on the one occasion?”*). Items 1 through 8 are rated on a five-point scale, which are scored from 0 (*Never*) to 4 (*Daily or almost daily*), whereas items 9 and 10 are rated on a three-point scale and are scored as 0 (*No*), 2 (*Yes, but not in the past*), and 4 (*Yes, during the past year*). The total score comprises the summing of each of the selected item scores. The total score can range from 0 to 40. A score of 8 or more indicates hazardous drinking. A score of 13 or more in women, and 15 or more in men, may indicate alcohol dependence. The AUDIT has demonstrated good reliability. For example, in a systematic review by Meneses-Gaya et al. [59] across ten studies the average Cronbach’s alpha was 0.80. In the present study, the general cohort and clinical cohort scale scores showed very good reliability with a Cronbach’s α of 0.85 and 0.95, respectively.

#### 2.2.9. Drug Abuse Screen Test-10 (DAST)

The ten-item DAST-10 [60] was used to assess drug use behaviours over the past 12 months (e.g., *“Do you feel bad or guilty about your drug use?”*). Each item is rated on a dichotomized scale (yes/no answers). Each “Yes” answer is scored with 1, while each “No” answer is score with 0—except for question 3 for which a “No” is scored with 1 while “Yes” is scored with 0. The total score ranges from 0 to 10: 0 = no problems; 1–2 = low problems; 3–5 = moderate problems; 6–8 = substantial problems; 9–10 = severe problems. In a systematic review on the psychometric properties of the DAS, Yudko, Lozhkina, and Fotus [61] found it to be a reliable measure with multiple studies citing a Cronbach’s α of over 0.90. In the present study, the general cohort and clinical cohort scale scores showed good reliability with a Cronbach’s α of 0.72 and 0.82, respectively.

#### 2.2.10. Brief Coping Orientation to Problems Experienced (Brief-COPE)

In order to assess coping behaviours individuals employed when experiencing stressful situations, the 30-item Brief-COPE was used [17]. Participants are asked to think about a recent stressful event in their life and how they coped within that situation. The Brief-COPE is rated on a four-point scale: 1 (*I haven’t been doing this at all*), 2 (*I’ve been doing his a little*), 3 (*I’ve been doing this a medium amount*), and 4 (*I’ve been doing this a lot*). The Brief-COPE has a total of 15 two-item subscales (e.g., self-distraction, substance use, humour). The subscale scores are then added together to give a score ranging from 2–8. A higher score on the subscale represents a higher utilisation of the related coping behaviour. These smaller subscales form a super-ordinate domain coping style. These are: emotion-focused coping (EFCope; scoring from 10 to 40), dysfunctional coping (DCope; scoring from 12 to 48), and problem-focused coping (PFCope; scoring from 6–24).

EFCope includes the subscales: Acceptance (e.g., learning to live with it), emotional support (e.g., comfort and understanding), humour (e.g., making jokes about it), positive reframing (e.g., look for something good in it), and religion (e.g., finding comfort in religious or spiritual beliefs). The scores of each subscale are summed and produce a total domain score from 10–40. The general and clinical cohorts showed good reliability with a Cronbach’s α of 0.83 and 0.91, respectively. DCope includes the subscales: Behavioural disengagement (e.g., giving up trying to deal with it), denial (e.g., saying to myself “this isn’t real”), self-distraction (e.g., turning to work or other activities to take my mind off things), self-blame (e.g., blaming myself for things that happened), substance use (e.g., using alcohol or other drugs to help me get through it), and venting (e.g., expressing negative feelings). The scores of each subscale are summed and produce a total domain score from 12–48. The general and clinical cohorts showed good reliability with a Cronbach’s α of 0.82 and 0.81, respectively. PFCope includes the subscales: Active coping (e.g., concentrating my efforts on doing something about the situation I am in), instrumental support (e.g., getting help and advice from other individuals), and planning (e.g., thinking hard about what steps to take). The scores of each subscale are summed and produce a total domain score from 6–24. In the present study, the general cohort and clinical cohort scale scores showed very good reliability with a Cronbach’s α of 0.80 and 0.88, respectively. 

As seen above, each of the domain subscales vary in subscales used and total. In order to control for this, each domain coping style was then transformed into a *z*-score scale of −2 to 2 so analyses could be carried out when comparing each domain.

### 2.3. Data Analyses

**Figure 1:**
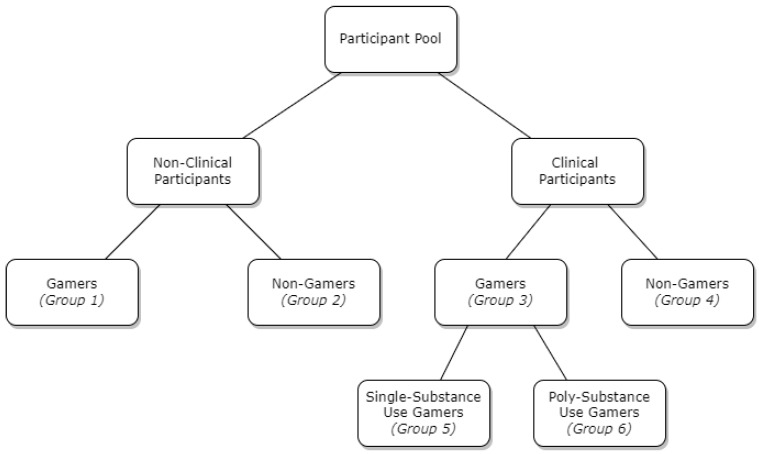
Participant groups: (1) gamers, (2) non-gamers, (3) clinical gamers, (4) clinical non-gamers, (5) single-substance use gamers, and (6) poly-substance use gamers.

Data were analysed using *Rstudio for Windows* (v3.5.1). Participants were categorised into six groups for analysis (as seen in Figure 1): (1) gamers, (2) non-gamers, (3) clinical gamers, (4) clinical non-gamers, (5) single-substance use gamers, and (6) poly-substance use gamers. In order to investigate H_1_, Spearman’s rho bivariate correlations (with Hochberg correction) were conducted to examine relationships between internet gaming characteristics, substance use variables (DAST and AUDIT scores were combined into a single substance use *z*-score). Chi-square analyses were then used to test for differences between single and poly-use gamers. In order to investigate H_2_, coping domain variables were calculated and standardised (*z*-score), and a multiple linear regression analysis was conducted to investigate the relationship of problem-focused, emotion-focused, and dysfunction coping on GD scores in both clinical and non-clinical gamers.

Following this, an exploratory comparison of clinical and non-clinical gamers was conducted to further investigate H_1_. The *z*-scores were calculated for the related variables (DCope, BYSAS, and IDS9) which were significantly correlated with the IGD9-SF. Non-clinical gamers were data-matched on age, qualification, and gender of the clinical gamers using the *Matchit* package in *Rstudio* [62]. During this process, any non-clinical gamer who scored 3 or higher on the DAST and/or 13 or higher on the AUDIT were removed from the potential pool, as they may be potential substance abusers. A series of *t*-tests were conducted to ensure that each gaming group did not significantly differ in age, qualification, or gender. Following this, the significant variables found among clinical and non-clinical gamers in H_1_ were then entered into a MANOVA to investigate the variance between the *z*-scores of clinical and non-clinical gamers, with appropriate follow-up post hoc tests.

## 3. Results

### 3.1. Correlations between Problematic Behaviours and Substance Use among Gamers and Non-Gamers

The results from the correlations between gamers’ scores and non-gamers’ scores on the respective scales are shown in Table 4. Correlation analyses showed that the gamers’ GD scores were significantly correlated with BYSAS and IDS9 scores with moderate effect sizes. The non-gamers showed a significant correlation between BYSAS scores and IDS9 scores with a moderate effect size [63]. Furthermore, gamers demonstrated more significant correlations overall than non-gamers.

When considering clinical gamers and clinical non-gamers, it was found that the clinical gamers’ GD scores correlated significantly with IDS9-SF scores with a strong effect size [63]. The clinical non-gamers showed significant correlations among BYSAS and BSMAS scores, and BSMAS and IDS9 scores with moderate effect sizes [63], the latter of which mirrored the clinical gamers. A full list of correlations between all the variables is shown in Table 5.

### 3.2. Co-Occurring Substance Use and Gaming among Clinical Gamers

A chi-square test was used to assess gaming behaviour and co-occurring drug use. The relation between these variables was significant χ^2^ (1, *n* = 64) = 1.01, *p* = 0.04 (with Hochberg correction), indicating that individuals who played videogames were more likely to use multiple substances (i.e., are poly-substance users). A *t*-test was then conducted to investigate if poly-substance users scored significantly differently on the IGDS9-SF scale when compared to single substance users. There was a significant mean difference of 8.4 (single-use *M* = 15.6; poly-use *M* = 24.09) in scores between single-substance users and poly-substance users, *t*(28.25) = −3.42, *p* < 0.01, indicating that participants who reported co-occurring drug use scored significantly higher on the IGDS9-SF.

### 3.3. The Effect of Coping Style on Gaming among Clinical and Non-Clinical Gamers

A multiple linear regression was calculated to predict GD scores based on three different coping styles: problem-focused coping (PFCope), emotion-focused coping (EFCope), and dysfunctional coping (DCope). In relation to non-clinical gamers, a significant relationship was found (*F* [3,83] = 3.61, *p* = 0.01), with an adjusted R^2^ = 0.08. The non-clinical gamers’ predicted gaming disorder scores are equal to 1.038 + 0.005 (DCope) + 0.007 (EFCope) − 0.009 (PFCope), where each was assessed on a scale. DCope was a significant predictor of gaming disorder scores (*p* = 0.01). In regard to the clinical gamers, a significant relationship was found (*F* [3,32] = 4.04, *p* = 0.01), with an adjusted R^2^ 0.20. The predicted GD scores were equal to 0.893 + 0.010 (DCope) + 0.008 (EFCope) − 0.008 (PFCope). DCope was a significant predictor of GD scores with a *p*-value of 0.01.

### 3.4. Exploratory Direct Comparisons of Clinical Gamers and Non-Clinical Gamers

A one-way multivariate analysis of variance was performed to determine the effect of gamer group on dysfunctional coping, gaming disorder, sex addiction, and internet addiction scores. There were three groups investigated: (i) gamers (non-clinical), (ii) single-use gamer (clinical), and (iii) poly-use gamer (clinical). There was a statistically significant difference between the groups on the combined dependent variables of dysfunctional coping, gaming disorder, sex addiction, and internet addiction scores (*F* [8,134] = 4.23, *p* < 0.001).

**Figure 2:**
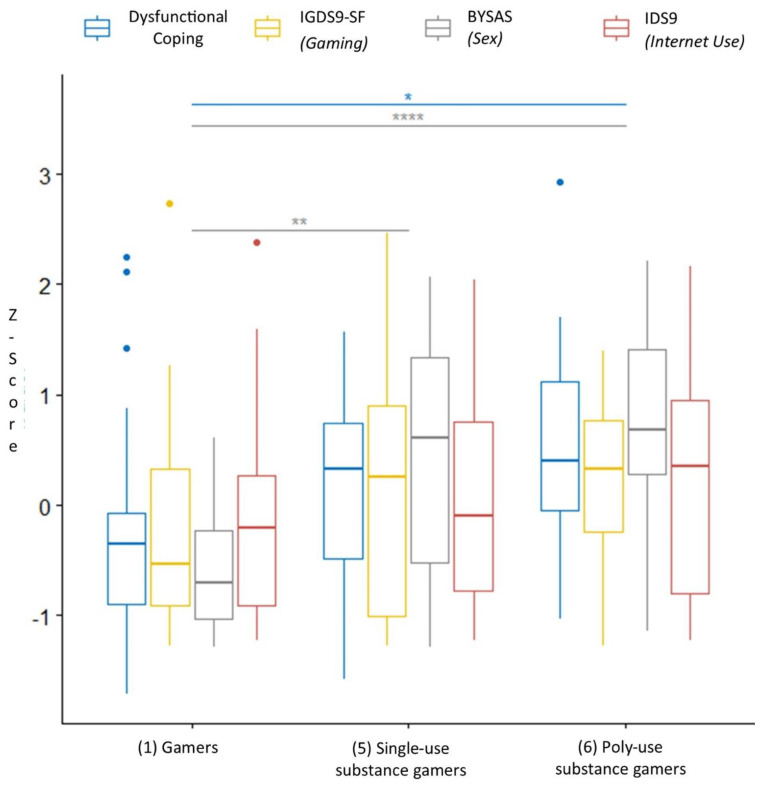
Pairwise Comparison. Games Howell pairwise comparison of *z*-scores with Tukey correction of group 1, 5, and 6. * *p* = 0.01; ** *p* = 0.003; **** *p* < 0.00014.

Follow-up univariate Welch ANOVAs, using Hochberg correction, showed that there was a statistically significant group difference in scores in dysfunctional coping (*F* [2,34.4] = 5.18, *p* = 0.03) and sex addiction using the BYSAS (*F* [2,28.5] = 19.2, *p* < 0.001). However, there were no significant differences between groups on gaming disorder scores (*F* [2,35.7] = 3.11, *p* = 0.11) or internet disorder scores (*F* [2,33.4] = 0.72, *p* = 0.49). Games Howell pairwise comparisons (adjusted Tukey *p*-value) were then conducted between the groups for each of the outcome variables, which can be seen in Figure 2. Significant differences were observed in dysfunctional coping between non-clinical gamers and poly-substance use gamers (*p* = 0.01), sex addiction scores between non-clinical gamers and single-substance use gamers (*p* = 0.003) and non-clinical gamers and poly-substance use gamers (*p* < 0.0001). Gaming disorder scores also approached significance between non-clinical gamers and poly-substance use gamers (*p* = 0.057).

## 4. Discussion

The present study compared and contrasted the co-occurring problematic behaviour and substance use among clinical and a non-/sub-clinical sample of gamers and non-gamers and explored the effect of coping strategies on gaming and its co-occurrence with substance use and other problematic behaviours.

### 4.1. Gaming Disorder, Problematic Behaviours, and Substance Use

In the present study, it was hypothesized that gamers would be more likely to exhibit co-occurring problematic behaviours among both clinical and non-clinical groups (H_1_). There were a number of significant correlations found within each of the groups. In regard to non-clinical gamers, it was found that GD scores were significantly positively correlated to internet addiction and sex addiction among gamers. This finding provides evidence consistent with previous observations within the field that GD is positively correlated with other problematic behaviours [10]. Moreover, within gamers, it was found that drug and alcohol use were also significantly correlated with sex addiction and problem gambling, aligning with the substance use literature [34]. This was not mirrored among the non-gamers, where there were no significant correlations with substance use. This finding suggests that gamers, when compared to non-gamers, may interact with substance use and related risky behaviours more than their non-gamer counterparts [10].

In regard to clinical gamers, the results suggest that GD scores were significantly correlated with internet addiction, social media addiction, and sex addiction, whereas the clinical non-gamers showed significant correlations between sex addiction and shopping addiction, and between internet addiction and social media addiction. This provides evidence which aligns with the known literature on GD and substance use, in which disordered behaviours (e.g., problematic videogame use) have been associated with increased substance use [30,33]. The similarities in correlations across gamers and clinical gamers appear to suggest that each group share defining characteristics in the way in which they engage with behaviours which may become problematic (e.g., sex). However, this needs to be investigated more thoroughly using a longitudinal design.

The results did not indicate any significant correlations between GD scores and substance use scores in either the non-clinical or clinical group. Previous research examining the correlations between substance use and GD has been somewhat mixed, as researchers have not found a consistent association between gaming and substance use [64]. While there have been a number of studies which consider videogames a risk factor for substance use, there are papers that have suggested gaming can act as a protective factor [65]. Recent research regarding gaming and substance use has suggested that the correlations between these two factors can vary depending on geographical location [66]. This may be a viable explanation as to why the present study did not find any significant associations between gaming and substance use specifically. To elaborate, it has been suggested that the association between gaming and substance use may be attributed to the perceived risk (i.e., the self-reported risk) of problematic gaming within the countries of which the samples were collected (i.e., Australia, New Zealand, or the United Kingdom). For example, the psychometric scales used in the present study required participants’ self-reports of problematic behaviours. However, the perceived societal acceptability of gaming is more favourable in western high-income countries [66], and therefore participants may view their gaming habits more favourably than habits related to substance use.

Consequently, this favourable view of gaming compared to substance use, such as alcohol use, may occur due to the relatively new nature of gaming. Societal attitudes towards who can consume alcohol, in which situations and contexts, to what amount, and the consequences for transgressing these social rules can vary from country to country [67]. Due to alcohol being consumed in countries all over the world for much of history [68], it, and its varying uses, have been rooted into the value framework (i.e., the values and views of citizens in a country) of the respective country [69]. Therefore, the societal acceptability of alcohol may refer to a nuanced attitude, in so far as that it has clear societal expectations and consequences (both social and legal) attached to it in a variety of settings, ranging from general use to problematic use. However, gaming lacks this nuanced understanding of the expectations and consequences (both social and legal, or lack thereof) within society, with no specific and clear values or views for the larger society to frame expectations and consequences. Therefore, when participants are requested to rate their own behaviour, they may compare it to the well-known expectations and risks of substance use (e.g., alcohol use) and inadvertently underestimate the perceived risk of gaming (or other problematic behaviours) [66].

Consequently, perhaps within the present sample, the geographical location, and the associated perceived risks held in that region (i.e., New Zealand), are reflected within self-reported results, in which they may perceive gaming is less risky than substance use. It is of course possible that the results suggest it is not the act of playing videogames that is related to substance use, but instead the similarities gamers may share with other at-risk populations (e.g., dysfunctional coping strategies) [10,27]. Indeed, it could be that co-occurring problematic behaviours or substance use, in conjunction with maladaptive coping strategies, create an exacerbating effect within gamers [24], which may be more pronounced in at-risk groups, such as substance users in abstinence [25].

### 4.2. The Exacerbating Effect of Co-Occurrence

In order to better understand the potentially exacerbating effect of co-occurring substance use (i.e., poly-use) in at-risk populations, the relationship between single-substance use and poly-substance use (i.e., co-occurring use) was explored in clinical gamers and non-gamers. The results suggest that the clinical gamers within the present sample were more likely to be poly-substance users when compared to clinical non-gamers who were likely to be single-substance users. Furthermore, a post hoc test indicated that within clinical gamers, there was a significant difference in GD scores between single-substance use gamers and poly-substance use gamers. Therefore, H_1_ was supported and provides evidence in line with the current literature, which suggests that there is a potential for problematic or disordered substance use to exacerbate other existing problematic behaviours [10,70]. The difference in scores may suggest that a cycle of reciprocity may exacerbate symptomology (as indicated by an increase in scores) between poly-substance users and abstinent single-substance users.

This is an important finding because it highlights the complexities of clinical symptomology, and the way in which co-occurring use may manifest other potential underlying risk factors [42]. Various studies have evidenced the co-occurrence of substance use and other addictive behaviours [37], and the present findings add to the literature suggesting that there can be overlaps in problematic/disordered behaviours and substance use. In line with this literature, it could be that the poly-substance users are at a higher risk of developing co-occurring at-risk behaviours, therefore resulting in a cycle of reciprocity [38,39,40]. In regard to gaming, there is a need for more research into the impacts of problematic gaming in abstinent substance-use populations [10]. The present study lends further support to at-risk populations having a higher tendency to develop problematic gaming behaviours [35,37]. Moreover, individuals who experience increased poly-substance use may also substitute maladaptive substances with potentially problematic behaviour [21].

Indeed, the findings, which show that abstinent poly-substance users score more highly than single-substance users on psychometric measures of problematic behaviours (e.g., sex addiction), lend support to replacement theory, wherein an individual who has stopped using one substance or behaviour (i.e., abstinence), may then become more vulnerable to other problematic behaviours [35]. This is partially due to exploration of other avenues to satisfy the behaviours’ specific function (e.g., coping). Moreover, when seeking to substitute these behaviours, the individual may choose behaviours which are perceived to be less detrimental. Consequently, it is possible that the higher scores achieved across a number of problematic behaviours in this group could indicate that these behaviours are perceived to be less detrimental to the original substance use or other potential substances [35]. This suggests that perceived risks of these behaviours are lower within the current clinical sample, aligning with the previous literature [66] and extending it to emerging adults and adults. The literature has also suggested that individuals in abstinence can adopt behaviours that can be repeated excessively in an attempt to self-regulate their mood state, therefore adopting it into their existing coping strategies [15,35].

### 4.3. Coping Strategies among Gamers

The present study explored three domains of coping to investigate the role coping played within gamers and clinical gamers. It was hypothesised that the severity of gaming among both clinical and non-clinical groups will be positively associated with dysfunctional coping styles, but negatively associated with problem-focused coping styles (H_2_). The results suggested that coping strategies among gamers were significantly associated with GD. More specifically, as hypothesised, dysfunctional coping strategies were significant indicators of higher GD scores for both gamers and clinical gamers. However, problem-focused coping, while it had a negative association with GD scores, it did not reach significance. Interestingly, the results demonstrated that coping strategies within gamers had a smaller association with GD (8%), although within the clinical gamers they had a stronger association (20%) with GD scores. The difference in these scores supports the notion that dysfunctional coping strategies may exacerbate problematic behaviours [21,25].

The present study’s findings are also in line with the literature on the relationship between gamers and their coping strategies [10], suggesting that there may be parallels in the way gamers and substance users regulate their emotions [27]. More specifically, the results suggest that some gamers develop dysfunctional coping strategies which manifest as excessive engagement in videogame play, therefore, indicating a maladaptive form of emotion regulation which is not seen when compared to gamers who have more varied and adaptive coping strategies and therefore emotion regulation strategies [71]. Maladaptive emotion regulation has been found to be associated with addictive behaviours and substance use [29]. Therefore, if abstinent substance users are gamers, and have developed dysfunctional coping strategies, they may engage in problematic gaming in order to replace (i.e., replacement theory) and/or regulate negative feelings or emotions (i.e., the self-medication hypothesis; [5,21]. The present findings suggest that poly-substance users may be at a higher risk of developing a repetitive use of videogames to achieve this goal, representing a unique risk factor within gamers abstaining from substance use. Therefore, it is important to consider coping strategies, their influence on emotion regulation, and the potential development and maintenance of co-occurrence in at-risk populations of gamers.

The findings also contribute to the broader literature on coping, suggesting that the avoidant behaviours within emotion-focused coping can be considered a third distinct dysfunctional coping domain. There has been research which has utilised the problem-focused and emotion-focused domains, with avoidant behaviours being included in the emotion-focused domain [15,22]. The dysfunctional coping domain has also been utilised in a number of studies which have considered avoidant emotion-focused coping styles as dysfunctional (e.g., denial, self-distraction) [21,72]. Therefore, the present study provides additional support for the consideration of a dysfunctional coping domain in the literature on coping. The use of an overarching dysfunctional coping domain could help in refining the understanding of emotion-focused coping through the separation of maladaptive emotion-focused styles (e.g., denial) and adaptive emotion-focused styles (e.g., emotional support). This is important, as when considering problematic and disordered behaviours, the way in which an individual approaches a stressful situation is a significant factor when considering their coping style [22]. While the overarching domains of problem-focused and emotion-focused strategies provide approach and avoidant style behaviours, respectively, a more nuanced approach on emotion-focused strategies may be useful [22]. Therefore, differentiating between adaptive emotion-focused coping (e.g., positive reframing) and maladaptive dysfunctional coping (e.g., self-distraction) is important when drawing parallels between separate populations who may share similarities in coping strategies as different populations may develop nuanced differences in their coping strategies.

### 4.4. Exploring Differences between Gamers and Abstinent Substance Use Gamers

The literature suggests that co-occurrence exacerbates disordered behaviours or substance use within individuals [10]. As such, the present study explored the way in which the co-occurrence of sex addiction, internet addiction, and/or coping styles may exacerbate existing problematic behaviours and/or substance use among gamers (non-clinical), single-substance use gamers (clinical), and poly-substance use gamers (clinical). As hypothesised, gamers with substance use history exhibited more dysfunctional coping compared to non-gamers with a history of substance use. More specifically, pairwise comparisons showed that dysfunctional coping scores were significantly different between gamers and poly-substance use gamers. Furthermore, there were significant differences in sex addiction scores between gamers and both single-substance use gamers and poly-substance use gamers. Finally, it was found that GD scores approached significance between gamers and poly-substance use gamers.

As previously discussed, dysfunctional coping is part of a maladaptive process which is associated with avoidant behaviour. When controlling for various demographic factors, there was a significant difference in coping scores between gamers and poly-substance use gamers, but not between gamers and single-substance use gamers. Therefore, it appears that avoidant behaviours are an important factor involved in the development of maladaptive coping mechanisms and can lead to adverse effects in poly-substance use gamers. The way in which coping strategies are used in different gamer populations may explain the difference in scores [10]. More specifically, gamers may interact and engage with stressors in a more active manner, whereas clinical gamers appear to be more likely to avoid the stressor. Previous literature pertaining to the self-medication hypothesis [28] can explain why this occurs among poly-substance users and why they may be more likely to engage in dysfunctional coping strategies. Specifically, when confronted with a stressful event, substance users may return to the substance or behaviour that alleviated the negative mood state effectively [28], therefore enforcing dysfunctional coping strategies. However, further research is needed within clinical populations of individuals who play videogames.

Within the present sample, there was no significant difference in technology-related disorders, such as problematic internet use or GD across groups. However, there were some interesting findings regarding sex addiction among gamers. Single-use gamers and poly-use gamers scored significantly higher in comparison to non-clinical gamers. Sex addiction has been found to be associated with individuals with disordered substance use and substance users in abstinence [35]. The present results align with this literature, demonstrating that sex addiction scores increased with substance use and poly-substance use when compared to those who do not engage with disordered substance use. This is a particularly interesting finding which lends support to replacement theory [35]. More specifically, the significant difference of sex addiction scores between each of the aforementioned groups could suggest that—within this particular sample—sexual activity may replace (or supplement the replacement of) a substance among abstinent users. Moreover, the increased sex addiction scores between single-substance use gamers and poly-substance use gamers lends support to the cycle of reciprocity, wherein the overlap of problematic behaviours creates a mutual exacerbation between two or more problematic behaviours [38,39,40]; therefore, suggesting that the co-occurrence of multiple addictive behaviours may be a viable explanation for the increase in sex addiction and substance use scores.

The culmination of these behaviours supports the present literature which suggests that co-occurring behaviours within GD and substance abuse may be enforced through dysfunctional coping strategies [25]. Furthermore, the present study offers novel insight into the effects of maladaptive coping strategies and the potential for co-occurrence to exacerbate disordered behaviour within a cohort of substance-abstinent gamers. What is less clear from these data is the extent to which gaming activities themselves may influence said behaviours among gamers. While it is possible that perceptions around videogame use may differ across studies due to the geographical location and wealth of the questioned sample, further multi-cultural data are required to investigate this theory.

### 4.5. Clinical Implications and Future Directions

The findings of the present study have implications for both clinical work as well as future research directions. The study expanded upon previous research on co-occurrence [34], coping [15], and utilised a clinical population sample, as recommended by previous research [10]. More specifically, the effect of co-occurring problematic behaviours on coping strategies has been highlighted, suggesting that dysfunctional coping strategies contribute to higher scores in gaming disorder, sex addiction, and internet addiction psychometric scores. This is an important finding as more research is needed to assess risk factors associated with disordered behaviour and the potential for co-occurring addictions [10]. This has practical clinical significance as it adds to the growing body of literature that suggests that underlying co-occurring disorders may require an integrated treatment approach [44,45]. Furthermore, the findings highlight the need for more research into the specific aspects of gaming, if any, which may facilitate co-occurrence. While the present results did not suggest that gaming scores were significantly different between gamers and poly-substance use gamers (post-transformation), it suggested that other behaviours such as problematic sexual activity may instead play a replacement role among gamers. While this falls in line with the substance use literature which suggests that substance users may replace substance use with other problematic or high-risk behaviours [35], future research should investigate problematic behaviours with larger clinical samples, examining a variety of substance uses with a focus on specific co-occurring usage, while considering the onset and length of use in relation to the development of other problematic behaviours.

Importantly, the present study further expands on previous work in regard to coping strategies [15,73,74], supporting the theory that dysfunctional coping styles exacerbate the association between psychopathological symptomatology and videogame use. Moreover, the present research extends these findings to a sample of gamers and substance-abstinent gamers. Therefore, the findings have implications for preventative efforts in abstinent substance use gamers and non-clinical gamers. For example, psychoeducation has been shown to be a useful tool for substance users in treatment as they can often lack insight into their symptomology [75]. Therefore, a psychoeducation approach could be utilized to aid substance use gamers in learning the risks of replacement behaviours and which coping styles are implicated in the process.

Understanding these connections has been shown to improve treatment outcomes, and should therefore be considered for clinic research and interventions [75]. Moreover, as a preventative measure, gamers who show signs of maladaptive coping strategies should be taught alternative approaches to deal with life stressors, as these may serve to increase their resilience. Future research could consider studying the over-time development and/or maintenance of these coping strategies, identifying specific gaming-related elements (e.g., playstyle or game genre) that may act as risk or resilience factors in the development of maladaptive coping. However, there are few relevant studies within the GD literature which consider clinical samples. Therefore, more research is needed with larger clinical samples to examine the effect of gaming-related factors on coping strategies and the resulting maladaptive psychopathology.

### 4.6. Limitations and Strengths

The present study contributes novel and insightful information concerning a little-studied cohort to a number of research areas. However, there are a number of limitations that should be considered when interpreting the results. The study employed self-report measures for both the general population and the clinical population. Participants may not accurately represent their behaviours related to substance use or problematic behaviours, which may lead to biased reporting. Furthermore, the present study’s sample size should be taken into consideration when interpreting the findings. Data for the clinical sample was collected using convenience sampling, and therefore may not be reflective of the wider population, meaning that these results may not be generalisable to the wider general populations or wider clinical populations. The sample sizes were small, which in part, was due to the availability of participants in the rehabilitation centre. In regard to the clinical cohort, when analysing data on co-occurrence, although participants self-reported multiple substance uses throughout a given day, it is possible substances were used consecutively and not in parallel. Therefore, caution is advised when extrapolating these results beyond the current sample. In addition, the cross-sectional nature of the present study means that no cause-effect conclusions can be made. Finally, there are some limitations of the brief-COPE measure as it assumes specific strategies will be consistently used, rather than the use of multiple different styles to deal with different stressors.

Despite this, the present study also has a number of notable strengths. There are few studies in the field which utilise substance abstinent gamers to study the potential of co-occurrence [10]. Therefore, the present study allows unique insights into an intersection of vulnerable populations (i.e., substance users and gamers). The present study also utilised a multifaced approach when comparing general gamers to clinical gamers, providing an insight into potential interactions and risks when engaging with problematic behaviours and substance use. Furthermore, the present study provides an important steppingstone for future research directions.

## 5. Conclusions

The aim of the present study was to investigate the coping styles and the co-occurrence of problematic/disordered gaming with other potentially addictive substance use and behaviours within clinical populations in treatment, an area which is in need of more empirical data [10]. Understanding how at-risk populations interact with potentially addictive behaviours and substance use while navigating life stressors can inform preventative strategies. As such, co-occurring problematic behaviour and substance use data was collected in both a clinical and a non/sub-clinical sample of gamers and non-gamers. These were then compared and contrasted based on the effect of coping strategies on gaming and its co-occurrence with substance use and other problematic behaviours. Furthermore, novel exploratory direct comparisons between the groups were also analysed.

The study’s findings suggest that gamers from different populations (i.e., general, and clinical) share similar at-risk behaviours. These problematic behaviours were more pronounced in abstinent substance use gamers, and more specifically poly-substance use gamers. The findings of the present study add to the literature which suggests that coping style and co-occurrence may have some impact on the assessment and potential treatment of substance abstinent gamers, offering support for an integrated treatment approach, wherein both substance use and the other problematic behaviours (e.g., gaming disorder) are considered in tandem [44]. Furthermore, gamers who do not meet a clinical criterion may also benefit from the development of new adaptive, problem-focused coping strategies to supplement or replace developing dysfunctional coping strategies [22,73].

## Figures and Tables

**Table 1:** Demographics and videogame use information (clinical group).

Sociodemographic Variables		Total (*n* = 64)
Gender	Male	49 (76.5%)
	Female	15 (23.4%)
Marital status	Same-sex civil partnership/married/Separated, but still legally in a same-sex civil partnership/married	6 (9.3%)
	Civil partnership has been dissolved/divorced	9 (14%)
	Never registered a same-sex civil partnership/married	32 (50%)
	Prefer not to say	17 (26.5%)
Qualification	Postgraduate degree (e.g., MA, PhD)/Degree (e.g., BA, BSc)	8 (12.4%)
	Professional qualification (e.g., teaching, nursing, accountancy)/Other vocational/work related qualifications	4 (6.4%)
	Foundation degree/Progression diploma/Advanced diploma/Certificate or equivalent	4 (6.4%)
	A levels/AS levels/VCEs/Higher diploma or equivalent/GCSEs/CSEs/O levels or equivalent	24 (37.4%)
	No qualifications or education/Prefer not to say	24 (37.4%)
Videogame use *	Yes	35 (54.7%)
	No	39 (45.3%)
	Years using playing videogames (*M* ± *SD*)	19.96 ± 6.76
	Hours spent playing videogames during a weekday (*M* ± *SD*)	4 ± 5.37
	Hours spent playing videogames during weekend day (*M* ± *SD*)	3.61 ± 3.53
Videogame platform *	Online PC games	3 (8.5%)
	Offline PC games	2 (5.7%)
	Online console games	7 (20%)
	Offline console games	8 (22.9%)
	Games for smartphones and tablets	15 (42.9%)

*Note.M* = mean *SD* = standard deviation; * denotes only applicable to those that answered “Yes” to videogame use; participants who listed that they play videogames were classified as gamers.

**Table 2:** Additional substance use information (clinical group).

Substance	How Many Times in the Last 30 Days? (in Days; *Mean* ± *SD*) *	Lifetime Use (in Years; *Mean* ± *SD*)	Route of Use **					Major Problem (Currently Abstaining) ***
			Oral	Nasal	Smoking	Non-IV Injections	IV Injections	
Alcohol—any use	3.4 ± 2.79 (*n = 5*)	16.04 ± 10.11 (*n = 58*)	92.7%	-	7.3%	-	-	46.9%
Alcohol—to intoxication	1 ± 0 (*n = 2*)	16.04 ± 10.11 (*n = 47*)	93%	2.3%	4.7%	-	-	N/A
Heroin	1 ± *N/A* (*n = 1*)	13.10 ± 13.81 (*n = 10*)	-	20%	30%	-	50%	3.1%
Methadone	-	8.16 ± 11.63 (*n = 12*)	36.4%	9.1%	-	-	54.5%	6.3%
Other (opiates/analgesics)	15 ± *N/A* (*n = 1*)	14.90 ± 12.43 (*n = 11*)	63.6%	9.1%	9.1%	-	18.2%	7.8%
Barbiturates	15 ± *N/A* (*n = 1*)	19.66 ± 12.57 (*n = 9*)	87.5%	12.5%	-	-	-	-
Other (sedative/tranquilizer)	5 ± 4.24 (*n = 2*)	17.11 ± 14.64 (*n = 9*)	66.7%	33.3%	-	-	-	3.1%
Cocaine	-	6.26 ± 9.53 (*n = 19*)	11.8%	58.8%	-	-	29.4%	1.6%
Amphetamines	3.21 ± 1.70 (*n = 4*)	12.8 ± 9.10 (*n = 50*)	15.2%	2.2%	58.7%	2.2%	21.7%	68.7%
Ecstasy	-	8.11 ± 8.08 (*n = 36*)	58.1%	19.3%	9.7%	3.2%	9.7%	4.7%
Cannabis	7 ± 12 (*n = 4*)	17.02 ± 10.79 (*n = 46*)	18.2%	81.8%	-	-	-	28.1%
Hallucinogens	-	12.76 ± 12.02 (*n = 21*)	82.6%	4.4%	13%	-	-	4.7%
Inhalants	4.5 ± 4.94 (*n = 2*)	7.90 ± 9.46 (*n = 11*)	54.5%	27.3%	18.2%	-	-	-
Nicotine	27.52 ± 5.77 (*n = 17*)	18.56 ± 11.22 (*n = 50*)	24.4%	75.6%	-	-	-	21.9%
More than one substance per day	9.6 ± 10.16 (*n = 5*)	16.62 ± 11.65 (*n = 24*)	28.6%	47.6%	-	-	23.8%	N/A

*Note.* * Of those not abstaining and of those who have used the indicated substance; ** Of those who have used indicated substance in their lifetime; *** Participants can be abstaining from more than one substance (*n* = 64); *SD* = standard deviation; N/A = not applicable.

**Table 3:** Demographics and videogame use information (control group).

Sociodemographic Variables		Total (*n* = 138)
Gender	Male	72 (54.5%)
	Female Other	59 (44.7%) 1 (0.8%)
Marital status	Same-sex civil partnership/married/Separated, but still legally in a same-sex civil partnership/married	29 (22%)
	Civil partnership has been dissolved/divorced	5 (3.8%)
	Widowed	1 (0.7%)
	Never registered a same-sex civil partnership/married	88 (66.7%)
	Prefer not to say	9 (6.8%)
Qualification	Postgraduate degree (e.g., MA, PhD)/Degree (e.g., BA, BSc)	55 (41.7%)
	Professional qualification (e.g., teaching, nursing, accountancy)/Other vocational/work related qualifications	5 (3.8%)
	Foundation degree/Progression diploma/Advanced diploma/Certificate or equivalent	8 (6.1%)
	A levels/AS levels/VCEs/Higher diploma or equivalent/GCSEs/CSEs/O levels or equivalent	54 (40.9%)
	No qualifications or education/Prefer not to say	10 (7.5 %)
Videogame use *	Yes	108 (81.8%)
	No	24 (18.2%)
	Years using playing videogames (*M* ± *SD*)	13.10 ± 7.51
	Hours spent playing videogames during a weekday (*M* ± *SD*)	4.24 ± 7.23
	Hours spent playing videogames during weekend day (*M* ± *SD*)	3.92 ± 3.73
Videogame platform *	Online PC games	18 (16.7%)
	Offline PC games	8 (7.4%)
	Online console games	26 (24.1%)
	Offline console games	21 (19.4%)
	Games for smartphones and tablets	35 (32.4%)

*Note.M* = mean *SD* = standard deviation; * denotes only applicable to those that answered “Yes” to videogame use.

**Table 4:** Spearman’s rho correlations of non-gamers and gamers in the general cohort.

	DA-Z	BYSAS	CDS	BSMAS	BSAS	IDS9-SF	PGSI
DA-Z		0.12	-	0.26	−0.04	0.01	−0.14
BYSAS	0.41 **		-	0.27	0.30	0.61 *	−0.40
CDS	0.52	0.18		-	-	-	-
BSMAS	0.03	0.19	−0.40		0.44	0.38	0.10
BSAS	0.16	0.26	0.07	0.47 **		0.29	0.29
IDS9-SF	0.10	0.46 **	0.08	0.53 **	0.41 **		−0.45
PGSI	0.30 *	0.13	-	0.17	0.26	0.14	
IGDS9-SF	0.04	0.39 **	0.21	0.21	0.16	0.59 **	0.14

*Note.* Non-gamers above the line and gamers below the line; non-gamers did not complete the IGDSF; CDS was n/a in the non-gaming group due to smokers’ *n* = 2; DA-Z—drug-use disorder/alcohol disorder *z*-score; BYSAS—Bergen-Yale Sex Addiction Scale; CDS—Cigarette Dependency Scale; BSMAS—The Bergen Social Media Addiction Scale; BSAS—Bergen Shopping Addiction Scale; IDS9-SF—Nine-item Internet Gaming Scale–Short Form; PGSI—Problem Gambling Severity Index; IDG9-SF—Nine-item Internet Gaming Disorder Scale–Short Form. * *p* ≤ 0.05; ** *p* ≤ 0.01.

**Table 5:** Spearman’s rho correlations of non-gamers and gamers in the clinical cohort.

	DA-Z	BYSAS	CDS	BSMAS	BSAS	IDS9	PGSI
DA-Z		0.13	0.17	0.25	0.03	0.16	−0.14
BYSAS	0.19		−0.16	0.20	0.69 **	0.36	0.24
CDS	−0.12	0.00		−0.01	0.25	0.69	−0.09
BSMAS	0.39	0.45	−0.07		0.25	0.67 **	−0.03
BSAS	−0.01	0.08	0.22	0.38		0.35	0.15
IDS9-SF	0.21	0.38 **	−0.04	0.70 **	0.46		−0.01
PGSI	0.57 **	0.34	0.23	0.52 *	0.17	0.27	
IGD9-SF	0.50	0.39 **	−0.07	0.62 **	0.16	0.71 **	0.37

*Note.* Non-gamer above the line and gamers below the line; non-gamers did not complete the IGDS9-SF; DA-Z—drug-use disorder/alcohol disorder *z*-score; BYSAS—Bergen-Yale Sex Addiction Scale; CDS—Cigarette Dependency Scale; BSMAS—The Bergen Social Media Addiction Scale; BSAS—Bergen Shopping Addiction Scale; IDS9-SF—Nine-item Internet Gaming Scale–Short Form; PGSI—Problem Gambling Severity Index; IDG9-SF—Nine-item Internet Gaming Disorder Scale–Short Form. * *p* ≤ 0.05; ** *p* ≤ 0.01.

## Data Availability

The data presented in this study are available on request from the corresponding author. The data are not publicly available due to the data being used in a larger doctoral project which is currently under examination.
